# Letter to the Editor: A List of New Group Divisible Designs

**DOI:** 10.6028/jres.096.037

**Published:** 1991

**Authors:** Kishore Sinha

**Affiliations:** Birsa Agricultural University, Department of Statistics, Ranchi-834006, India

**Keywords:** experimental design, group divisible design, partially balanced designs

## Abstract

Group divisible designs are the most important class of partially balanced incomplete block (PBIB) designs. A list of new group divisible designs with *r*, *k* ⩽ 10 is provided.

## 1. Introduction

Group divisible designs are the most important class of partially balanced incomplete block (PBIB) designs. Clatworthy, Cameron, and Speckman at the National Bureau of Standards [3] tabulated group divisible designs with *r*, *k* ≤10. Since then, a good number of new group divisible designs not listed in reference [3] have been reported by various authors in different journals. Here, a list of new group divisible designs not found in the tables in reference [3] is provided.

## 2. New Group Divisible Designs

Table 1 gives new group divisible designs with *r*, *k* ⩽ 10.

## Figures and Tables

**Table 1:** New group divisible designs with *r*, *k* ⩽ 10.

No.	*v*	*r*	*k*	*b*	*m*	*n*	λ_1_	λ_2_	E^a^	Source^b^
1	16	9	3	48	8	2	4	1	0.69	BP[1]
2	18	10	3	60	9	2	4	1	0.69	F[7]
3	12	7	4	21	6	2	1	2	0.82	F[7]
4	12	7	4	21	2	6	3	1	0.79	JT[8]
5	12	8	4	24	4	3	3	2	0.82	JT[8]
6	12	9	4	27	2	6	3	2	0.82	JT[8]
7	12	9	4	27	6	2	7	2	0.75	F[7]
8	12	10	4	30	6	2	0	3	0.81	F[7]
9	14	10	4	35	7	2	6	2	0.80	F[7]
10	16	6	4	24	8	2	4	1	0.78	BP[1]
11	18	10	4	45	6	3	0	2	0.80	F[7]
12	20	8	4	40	10	2	6	1	0.76	BP[1]
13	22	8	4	44	11	2	2	4	0.77	F[7]
14	24	9	4	54	12	2	5	1	0.77	F[7]
15	26	10	4	65	13	2	6	1	0.76	F[7]
16	14	10	5	28	7	2	4	3	0.86	JT[8]
17	15	8	5	24	3	5	3	2	0.86	JT[8]
18	15	8	5	24	5	3	4	2	0.85	JT[8]
19	15	10	5	30	3	5	5	2	0.84	S[10]
20	22	10	5	44	11	2	0	2	0.84	F[7]
21	12	7	6	14	6	2	5	3	0.91	JT[8]
22	12	9	6	18	6	2	5	4	0.91	F[7]
23	12	9	6	18	3	4	7	3	0.89	BP[2]
24	12	10	6	20	3	4	6	4	0.91	JT[8]
25	16	9	6	24	4	4	7	2	0.86	S[10]
26	12	7	7	12	3	4	6	3	0.92	BP[2]
27	16	7	7	16	4	4	2	3	0.91	JT[8]
28	16	7	7	16	8	2	0	3	0.91	D[4]
29	21	7	7	21	7	3	3	2	0.90	F[7]
30	24	7	7	24	8	3	0	2	0.89	F[7]
31	35	7	7	35	7	5	3	1	0.87	F[7]
32	45	7	7	45	15	3	0	1	0.88	DR[6]
33	42	8	8	42	7	6	4	1	0.88	F[7]
34	16	9	9	16	4	4	4	5	0.95	JT[8]
35	18	10	9	20	3	6	4	5	0.79	JT[8]
36	20	9	9	20	4	5	3	4	0.94	JT[8]
37	20	9	9	20	10	2	0	4	0.93	D[4]
38	24	9	9	24	6	4	4	3	0.93	S[9]
39	38	9	9	38	19	2	0	2	0.91	DR[6]
40	40	9	9	40	10	4	0	2	0.91	DN[5]
41	49	9	9	49	7	7	5	1	0.89	F[7]
42	21	10	10	21	7	3	9	4	0.94	F[7]
43	21	10	10	21	3	7	8	3	0.93	BP[2]
44	24	10	10	24	8	3	3	4	0.94	S[9]
45	28	10	10	28	7	4	6	3	0.93	F[7]
46	56	10	10	56	7	8	6	1	0.89	F[7]

a E stands for average efficiency factor.

b The abbreviations BP, D, DN, DR, F, JT, and S stand for Bhangwandas and Parihar, Dey, Dey and Nigam, De and Roy, Freeman, John and Turner, and Sinha, respectively.
